# Development of a COVID-19 Web Information Transmission Structure Based on a Quadruple Helix Model: Webometric Network Approach Using Bing

**DOI:** 10.2196/27681

**Published:** 2021-08-26

**Authors:** Yu Peng Zhu, Han Woo Park

**Affiliations:** 1 Blockchain Policy Research Center Cyber Emotions Research Institute Yeungnam University Gyeongsan-si Republic of Korea; 2 Department of Media and Communication Yeungnam University Gyeongsan-si Republic of Korea; 3 Interdisciplinary Graduate Programs of Digital Convergence Business Yeungnam University Gyeongsan-si Republic of Korea; 4 Interdisciplinary Graduate Programs of East Asian Cultural Studies Yeungnam University Gyeongsan-si Republic of Korea

**Keywords:** quadruple helix model, COVID-19, structural analysis, content analysis, network analysis, public health, webometrics, infodemiology, infoveillance, development, internet, online health information, structure, communication, big data

## Abstract

**Background:**

Developing an understanding of the social structure and phenomenon of pandemic information sources worldwide is immensely significant.

**Objective:**

Based on the quadruple helix model, the aim of this study was to construct and analyze the structure and content of the internet information sources regarding the COVID-19 pandemic, considering time and space. The broader goal was to determine the status and limitations of web information transmission and online communication structure during public health emergencies.

**Methods:**

By sorting the second top-level domain, we divided the structure of network information sources into four levels: government, educational organizations, companies, and nonprofit organizations. We analyzed the structure of information sources and the evolution of information content at each stage using quadruple helix and network analysis methods.

**Results:**

The results of the structural analysis indicated that the online sources of information in Asia were more diverse than those in other regions in February 2020. As the pandemic spread in April, the information sources in non-Asian regions began to diversify, and the information source structure diversified further in July. With the spread of the pandemic, for an increasing number of countries, not only the government authorities of high concern but also commercial and educational organizations began to produce and provide significant amounts of information and advice. Nonprofit organizations also produced information, but to a lesser extent. The impact of the virus spread from the initial public level of the government to many levels within society. After April, the government’s role in the COVID-19 network information was central. The results of the content analysis showed that there was an increased focus on discussion regarding public health–related campaign materials at all stages. The information content changed with the changing stages. In the early stages, the basic situation regarding the virus and its impact on health attracted most of the attention. Later, the content was more focused on prevention. The business and policy environment also changed from the beginning of the pandemic, and the social changes caused by the pandemic became a popular discussion topic.

**Conclusions:**

For public health emergencies, some online and offline information sources may not be sufficient. Diversified institutions must pay attention to public health emergencies and actively respond to multihelical information sources. In terms of published messages, the educational sector plays an important role in public health events. However, educational institutions release less information than governments and businesses. This study proposes that the quadruple helix not only has research significance in the field of scientific cooperation but could also be used to perform effective research regarding web information during crises. This is significant for further development of the quadruple helix model in the medical internet research area.

## Introduction

### Background

Since the first reported case of COVID-19 in late 2019, the disease rapidly spread to become a pandemic in March 2020. An infectious disease caused by a pathogen generally spreads to a living host, and it is easily transferable from the infected. As of January 7, 2021, over 87 million people have been infected with the virus in 190 countries and regions worldwide since the outbreak of COVID-19 in February 2020, resulting in a global catastrophe [1].

Social disasters, including infectious diseases, must be controlled through a process of actual data–based analysis [2]. Real-time assessment is critical for disaster monitoring; special attention must be paid to rapid analysis using relevant social and cultural data at both the macro and micro scales. The core of sociocultural data analysis is to understand, identify, and even predict the risk of transmission. Thus, a system needs to be constructed to collect data based on the disaster type or area, and to support the spontaneous decision-making process. Without this system, the public would face an information overload, as they would feel burdened dealing with an enormous amount of information in critical situations [3].

In today’s knowledge-based society, information production goes beyond traditional media organizations and involves many entities. In particular, with the increased popularity of smartphones, the amount of online information produced by individuals and organizations has grown exponentially. As the information production process becomes increasingly complex, traditional media companies face a situation in which the so-called legacy media’s usage time has decreased and is now competing with various sources [4]. Search engines and web portals are catering to a wider range of user needs than traditional alternatives [5]. Low cost is an important reason that internet information channels have more advantages than traditional information channels [6]. This phenomenon is especially obvious when significant events occur. The same is true for the information about COVID-19. Fear, anger, and other emotions also lead people to believe and spread online information available through nontraditional media, regardless of whether it is fake [7]. This implies that not only individual media and informal organizations but also a large number of formal organizations such as governments, academic institutions, and formal public organizations have started using online media to produce and disseminate information. However, sensitivity to fake news is also often influenced by political ideology [8]. In some countries, citizens do not support direct government control of the news, and are more concerned with information sources in cooperation with the media and other nongovernmental organizations [9]. Therefore, web media represent a strong competitor to traditional media in terms of both production and services.

COVID-19 has had a strong impact on the media system [10-12]. People have created significant numbers of online documents by utilizing new media sources such as Facebook and Twitter [13-15]. In fact, governments in some countries have used these forms of new media to build platforms to help combat the virus [16]. For example, due to the COVID-19 outbreak in Wuhan in February 2020, and the shortage of medical resources and services, Weibo, one of the largest new media companies in China, cooperated with the local government to set up a citizen assistance platform on which citizens with real-name identification could ask for help. This new mode of interaction is difficult to create with traditional media. To deal with the COVID-19 pandemic that is currently threatening the world, it is evident that the greater the number of internet information sources, the stronger the social immune system.

Therefore, in this study, we collected information sources with high online presence through big-data techniques in countries with confirmed COVID-19 cases. Additionally, the information related to COVID-19 in three stages (from February to April to July 2020) was analyzed in detail, and the morphology and trend of the web big data in these first 6 months of the pandemic are discussed. From these large-scale online big data, the information dissemination trends of educational institutes, enterprises, and government, and their contents were analyzed. In the case of an emergency or a disaster, this study can systematically explain the multielement spiral structure of the information transmission source in the cyberspace of major countries, which has high academic and social value.

In general, based on the quadruple helix and network analysis method, this study constructed and analyzed the structure and content of internet information sources of COVID-19 considering time and space. The aim was to determine the status and limitations of web information transmission and online communication structure in public health emergencies. Moreover, based on the content revealed, valuable suggestions are proposed to contribute to the internet communication of future public health events.

### Online Information Sources

With the rapid growth of the internet, web data analysis (often called “webometrics”) has become important, and its quantitative vastness and content diversity have been increasing accordingly. As mobile phones, tablets, and other mobile terminals have been growing in popularity, people usually use these terminals to obtain information instead of traditional media. Although some mainstream media outlets have their own web feeds, people generally use digital feeds from search engines such as Bing or Google to obtain information. Therefore, the study and analysis of comprehensive network information is often more objective than the study of specific news media, and more comprehensive information can be collated. For example, Thelwall [17] used Wikipedia data collected by Bing to study public interest in astronomy. Park et al [18] used Twitter and YouTube to analyze the spread of the Occupy Wall Street movement. Park and Lim [19] analyzed North Korean propaganda changes using YouTube media data. Cho and Park [16] used network activity information about the agriculture, forestry, and fishery departments to discuss the use of internet innovation by government organizations. This literature indicates that analysis and research on web sources have drawn useful conclusions in many areas to date.

There has also been substantial research regarding online sources of information at the time a disaster occurs. Jung and Park [20] used webometric methods to track and analyze the information networks of various organizations during the Gumi chemical spill in South Korea. They found that the flow of information between agencies had an impact on mobilizing emergency facilities and planning specific emergency responses. Online sources of information can also help alleviate the damage caused by disasters. Allaire [21] studied the Bangkok floods and found that social media users were obtaining real-time updates that could help to reduce their losses. Park [22] analyzed YouTube social activities during the 2016 South Korean earthquake, and found that YouTube became a channel to raise public crisis awareness and promote safety strategies. Kim et al [23] also elaborated on the role of social media in relaying information during disasters. By studying data from online information sources during the 2017 storms in the United States, it was found that the flow of information across the network was controlled by many types of users. Song et al [24] studied the differences and the range of emotions people felt toward local online channels, including publishing boards, Twitter, cafes, blogs, and news, that delivered information related to MERS (Middle East Respiratory Syndrome) [24]. Some scholars also analyzed the network information source data regarding COVID-19. For example, Park et al [25] collected Twitter data and found that monitoring public dialog and rapidly spreading media news can help professionals make complex and rapid decisions. However, web page data in internet information sources are often more stable than those in a social media environment [26]; therefore, we adopted web page data for analysis.

To grasp and respond to the situation of worldwide disasters such as COVID-19, we collected and analyzed the network information source data of countries with a large number of confirmed cases to understand how the disaster-related information provided by multiple sources changes over time. We identified three time periods (February, April, and July 2020), and performed a detailed analysis of the differences in information sources at these times. We also studied the structure of national cyberspace information sources. Through these analyses, we identified the structural evolution of web information publishers and clearly revealed the dynamic changes of information content in each period.

### Research Questions

Our primary research questions were as follows: (1) What are the structures and form of the COVID-19 web-mediated network among countries? (2) Are there differences in the keywords and topics of COVID-19–related online information at different stages?

## Methods

### Data Collection

Data were collected using Webometric Analyst 4.1 through Bing, which is one of the most widely used search engines that is available in most countries and regions, including Mainland China. In addition to Google, the Bing search engine is also often used to carry out scientific research [27-29]. Although Google is the world’s largest search engine, it is not available in some regions, including mainland China. Since web page data from China were very important for this study, we used the Bing platform for data collection. Website and domains obtained through the search application programming interface service of Bing were analyzed. In February 2020, the most widely used COVID-19 keyword in the world was “coronavirus.” Therefore, the keyword for the data collected on February 12 was “coronavirus.” After February, the terms “COVID-19” and “2019-nCOV” were also widely used. Therefore, we chose “COVID-19 OR Coronavirus OR 2019-nCOV” for the keyword searches performed on April 17 and July 22, 2020. Data were collected in real time, instead of collecting all information during a certain period. In other words, the data for these three time points (February 12, April 17, and July 22, 2020) are the results of real-time relevant searches on Bing for that day. Instant messages were not limited to the time of publication, and they may contain previously published information that is still highly popular. In addition, instant messages can reflect the actual state of the internet data at that time.

The time of the first collection was in the initial stage of the outbreak, the time of the second collection corresponded to the stage at which the number of new diagnoses had leveled off after spreading worldwide, and the time of the third collection corresponded to the stage when the number of newly diagnosed patients increased sharply as a second wave. For February, we collected data for all 28 countries and regions with confirmed cases, with a total of 9149 data points. For April 17, we collected data from 29 countries and regions with over 7000 confirmed cases and obtained 14,768 data points. For July 22, we collected data of over 70,000 people who received a diagnosis in 30 countries and regions, and obtained 14,483 pieces of data. Our data were collected from the top countries with the highest number of diagnoses per stage, not only from English-speaking countries. To ensure consistency, we only analyzed the English data on the webpage.

### Quadruple Helix

Quadruple helix is a research method based on triple helix, which was proposed in 1995 [30]. Researchers studied the development of the knowledge-based economic structure through the spiral relationships among universities, industries, and the government. At first, the triple helix model was used to explain the interaction among academia, government, and industries, and was often used in research related to knowledge production [31]. However, with the development of the triple helix theory, more elements were considered. In 2009, Carayannis and Campbell [32] introduced elements representing the public into the spiral model, such as the civil society and media, thereby forming a quadruple helix model; they added a research method at the level of new technologies and social needs. In 2010, Carayannis and Campbell [33] added the natural environment factor and constructed the quintuple helix model. Based on this factor, the relationship between innovation and sustainable development can be discussed. During cooperation and communication between various organizations, when one kind of organization occupies a dominant position, it can be considered that this organization is separated from the collection of various organizations, and the relationships among different organizations can be studied and explained through the quadruple, quintuple, or n-tuple helix concept [31]. This spiral structure does not always exist only in the academic, government, and industrial dimensions.

We collated the second top-level domain (TLD) data, which were categorized as data from commercial organizations, educational institutes, governments, and nonprofit organizations. A total of 38,399 domains were collected. To better classify the effective levels, we first sorted all of the collected second TLD data. After frequency analysis, the second TLD data that had an occurrence frequency higher than 1% (91/9149; 148/14,767; 145/14,483) were selected for classification. From 15,813 data units, we extracted four levels, namely governments, commercial enterprises, educational institutes, and nonprofit organizations. We used the quadruple helix model to analyze the structural dynamics of the four institutional levels at different periods in detail. For convenience in figures, we abbreviate government domains such as “.gov,” “gob,” and “.go” collectively as “G” (governments); educational domains such as “.edu” and “.ac” as “E” (educational institutes); commercial domains such as “.com” and “.co” as “C” (commercial organizations); and “.org” and “.or” nonprofit domains as “O” (nonprofit organizations).

### Network Analysis

A network analysis method was used to analyze the structure of the quadruple helix in detail. Network analysis is a method of quantitative analysis of nodes and connections in a network. When individuals and organizations act as nodes, the connection between them acts as a link. Through the quantitative results of the structure, the characteristics and nature of the network composed of these entities can be analyzed [34]. Network analysis has been widely used in social science research such as in social media use, knowledge dissemination, and organizational cooperation [35,36]. Although nodes have the same properties in a one-mode network, nodes differ in two-mode networks. Thus, in the one-mode network, nodes are the institutional components of a quadruple helix: government, private/business, educational institutions, and nonprofit organizations. By contrast, the two-mode network focuses on the relationship between the analyzed countries and the four institutional types. Centrality indices are important quantitative indices in network analysis, including degree centrality, betweenness centrality, eigenvector centrality, closeness centrality, and others [37-40]. The centrality index used in this study was degree centrality. Degree indicates the direct relationship between the nodes [39,40]. In this study, the degree was mainly used to determine whether the governments, education institutions, nonprofit organizations, and commercial organizations of different countries have similar information, and to observe the helix degree of different countries and the four fields. Although betweenness centrality is an important indicator for evaluating the influence of a mediating effect, the link between countries in this study is common information without mediating phenomena; thus, it was not used for this analysis. In addition, the eigenvector centrality is an index to evaluate the importance of each node connected to other nodes. However, in this study, based on the importance of government, education, public authorities, and companies, it was considered to be less important to evaluate the significance of the connected countries’ eigenvector centrality. Closeness centrality is an indicator of the shortest distance, and was also considered to be of little significance for this study. However, the degree centrality index can judge the strength of direct connections between countries and domains. In other words, the more countries connected in a field, the stronger the influence of this field. Therefore, only degree centrality was used for this analysis.

We used UCINET6 for network analysis and network visualization, including triple helix network analysis and content analysis. In the content analysis, we used the convergence of iterated correlations (CONCOR) method to cluster the semantic network in which words are regarded as nodes and cooccurrence between words forms a tie. CONCOR is a method of performing repeated cross-node correlation analyses to identify the appropriate level of similarity [41]. In other words, we first organized the relationships among words into matrices, thus forming a network of relationships among words. We then calculated the correlation coefficients between the rows and columns in the matrix and carried out the same calculation for the obtained correlation coefficient matrix. After repeated calculations, a correlation coefficient matrix consisting of only 1 and –1 was obtained, which was thus divided into two categories. We then performed the same calculation for both categories again and obtained four different clusters.

## Results

The hit counts and domains for each country (or region) are compiled and listed in Tables 1-3 for the three time periods, respectively. We standardized the number of hits and domain names for the three months (February, April, and July), with the maximum value set to 100 and the rest being the ratio of the original value to the maximum value multiplied by 100.

**Table 1:** Hit counts and domains for February (N=28).

Country	Hit counts	Domains
Australia	100.00	100.00
Canada	32.46	53.23
Italy	24.69	67.93
United Kingdom	23.93	33.08
Germany	21.05	43.98
France	18.30	33.46
Spain	13.66	44.61
Belgium	10.41	38.91
Japan	7.29	81.62
India	5.99	33.33
Mainland China	5.61	47.40
Singapore	5.34	69.84
Malaysia	4.10	28.77
United States	3.11	64.39
Hong Kong (China)	2.26	68.44
United Arab Emirates	2.22	7.73
South Korea	1.63	32.07
Taiwan	1.29	35.61
Philippines	1.29	29.91
Sweden	1.10	58.17
Sri Lanka	0.83	10.65
Vietnam	0.70	40.18
Finland	0.61	19.90
Russia	0.42	47.02
Thailand	0.18	41.70
Macau	0.16	14.45
Nepal	0.06	5.83
Cambodia	0.02	7.35

**Table 2:** Hit counts and domains for April (N=29).

Country	Hit counts	Domains
Canada	100.00	81.26
France	69.96	79.83
England	61.98	58.84
Germany	53.23	83.51
Brazil	51.71	88.14
Italy	46.01	75.92
US	32.78	99.88
Sweden	31.67	100.00
Belgium	30.61	44.13
Japan	30.42	61.80
Austria	21.48	41.04
Netherlands	20.95	93.24
Spain	18.17	59.43
Turkey	18.10	49.23
Chile	16.73	55.63
India	16.35	43.06
Switzerland	13.92	67.50
Denmark	13.50	84.82
Russia	9.16	27.05
Portugal	9.13	54.09
Ireland	8.33	45.20
Korea	6.39	46.74
Peru	5.86	31.55
Mainland China	4.18	35.11
Poland	3.84	75.56
Romania	3.31	49.11
Ecuador	2.95	41.28
Israel	2.67	41.64
Iran	0.36	46.38

**Table 3:** Hit counts and domains for July (N=30).

Country	Hit counts	Domains
United Kingdom	100.00	3.18
Canada	47.74	87.18
France	46.76	81.88
Mainland China	36.35	49.18
Brazil	33.40	92.00
Germany	32.22	4.59
Italy	24.56	94.24
Argentina	15.11	81.06
Mexico	13.67	89.06
Spain	11.00	4.47
South Africa	8.47	57.65
India	7.52	84.82
Turkey	7.43	71.41
United States of America	6.35	3.41
Russia	6.25	81.76
Colombia	4.20	68.00
Peru	4.01	41.18
Sweden	3.61	83.88
Chile	2.85	77.53
Ecuador	1.80	58.59
Indonesia	1.53	100.00
Pakistan	1.07	57.65
Philippines	1.02	61.41
Egypt	0.73	30.59
Iran	0.57	58.47
Bangladesh	0.47	42.47
Kazakhstan	0.26	44.82
Saudi Arabia	0.15	32.94
Qatar	0.05	35.06
Iraq	0.02	25.41

Table 1 shows that the countries with the highest hit counts in February were Australia, Canada, Italy, the United Kingdom, and Germany. Table 2 shows that the countries with the highest hit counts in April were Canada, France, and the United Kingdom. Table 3 shows that the countries with the highest hit counts in July were the United Kingdom, Canada, and France. In February, there were more domains in Australia, Japan, and Singapore. In April, more domains were observed in Sweden, the United States, and the Netherlands. In July, more domains were found in Indonesia, Italy, and Brazil.

**Figure 1:**
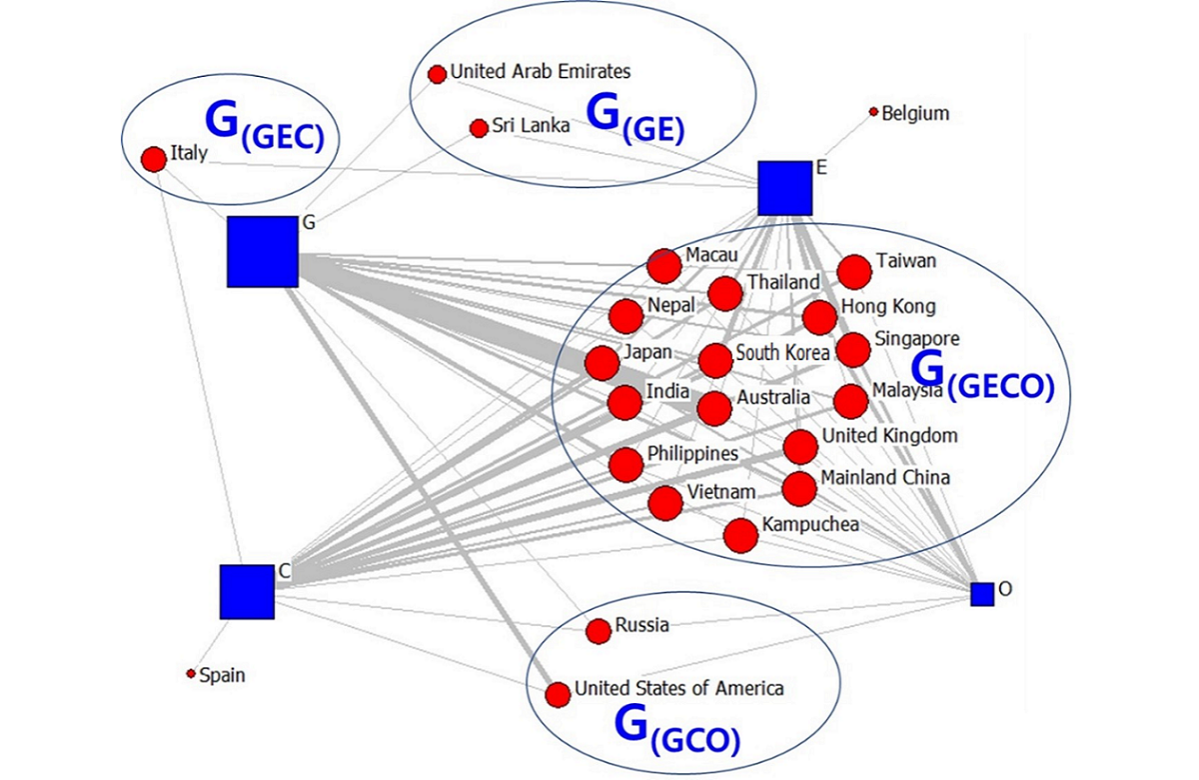
Two-mode quadruple helix structure in February 2020. Large "G" refers to the group. G: government domains; E: educational institute domains; C: commercial domains; O: nonprofit organization domains.

**Figure 2:**
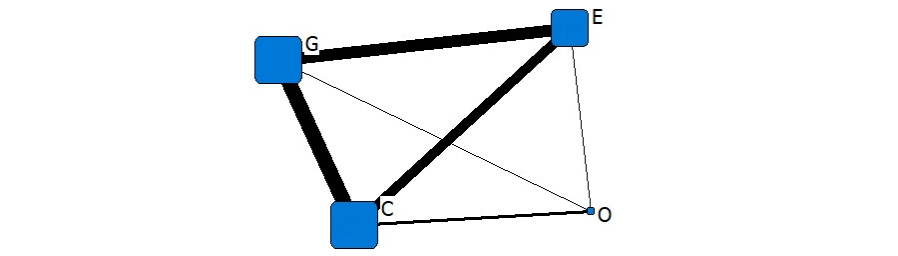
One-mode quadruple helix structure in February 2020. G: government domains; E: educational institute domains; C: commercial domains; O: nonprofit organization domains.

**Figure 3:**
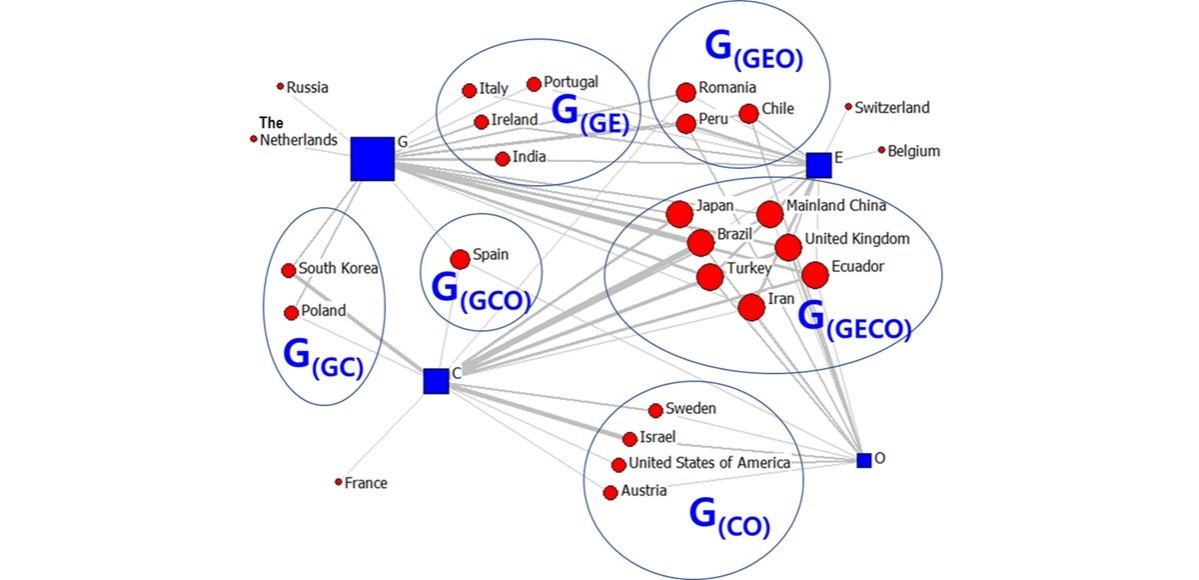
Two-mode quadruple helix structure in April 2020. Large "G" refers to the group. G: government domains; E: educational institute domains; C: commercial domains; O: nonprofit organization domains.

**Figure 4:**
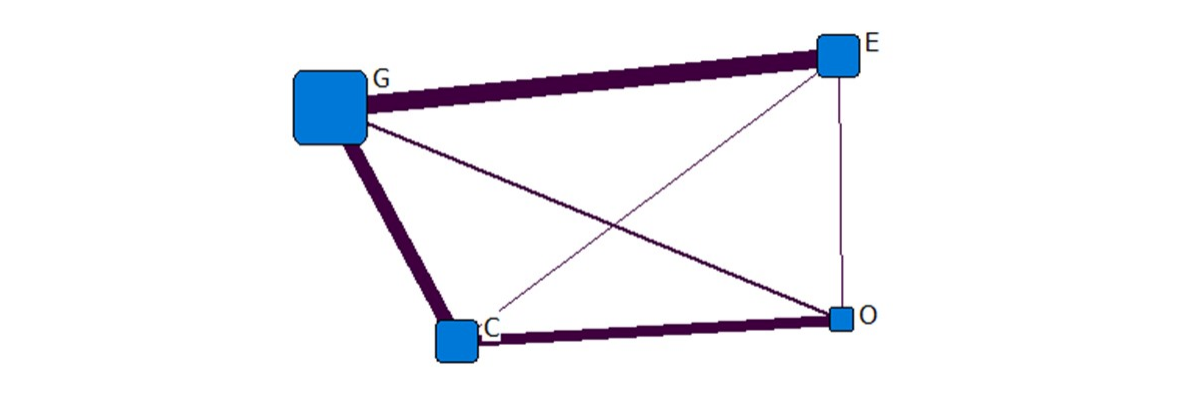
One-mode quadruple helix structure in April 2020. G: government domains; E: educational institute domains; C: commercial domains; O: nonprofit organization domains.

**Figure 5:**
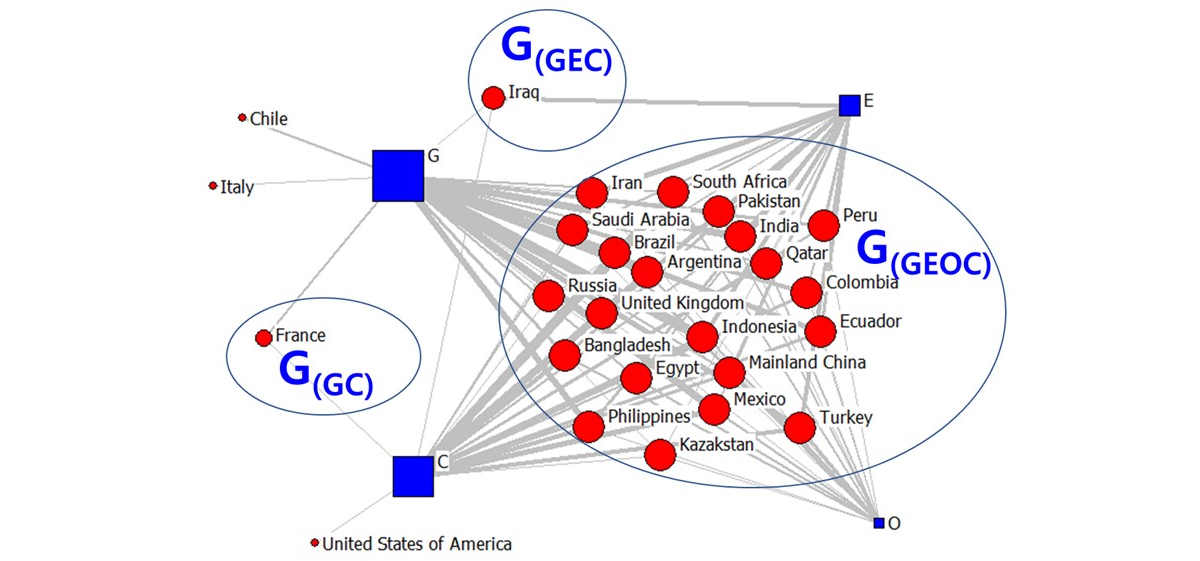
Two-mode quadruple helix structure in July 2020. Large "G" refers to the group. G: government domains; E: educational institute domains; C: commercial domains; O: nonprofit organization domains.

**Figure 6:**
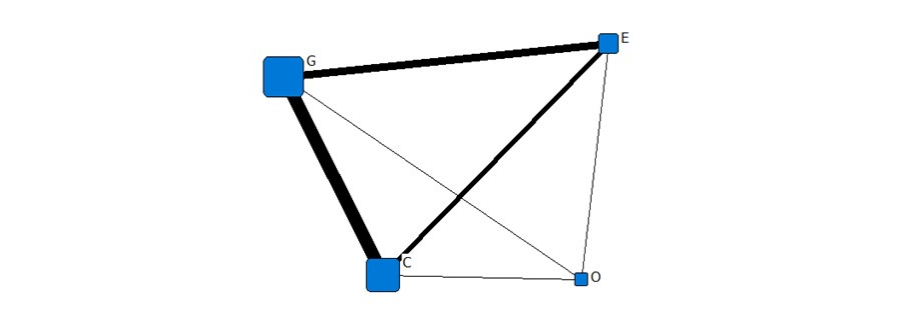
One-mode quadruple helix structure in July 2020. G: government domains; E: educational institute domains; C: commercial domains; O: nonprofit organization domains.

Following these results, we analyzed publishing organizations. We visualized the countries involved in the high-frequency second TLD data as a two-mode network. The data revealed 23 countries in February, 26 countries in April, and 26 countries in July. The visualization results are shown in Figures 1-6. The large “G” in the figures denotes “group.” For example, G(GEOC) means the group in which G (government), E (education), O (nonprofit organizations), and C (commercial organizations) appear simultaneously.

As seen in Figure 1, most of the COVID-19–related messages released in February were from the government, educational, or commercial sectors, with relatively few messages from the nonprofit sector. We divided the countries into several groups based on areas. Among all groups, the countries and regions that received information from these four areas the most included mainland China, Hong Kong, Macao, Australia, and Vietnam. Asian countries accounted for 88% (14/16) of these countries. Information from Italy was primarily from the government and educational enterprises. In Sri Lanka and the United Arab Emirates, information was mainly from the government and educational institutes. In the United States and Russia, information was from the government and the educational and commercial sectors. In the United States, government agencies were primarily concentrated in California. Spain reported more commercial agencies, whereas Belgium reported the most information from the educational field. In general, Asian countries were more diverse regarding the online information shared on COVID-19 in February than other regions. This could be because most of the confirmed COVID-19 cases were diagnosed during this period in Asia, and various regions of the continent were considered to be more sensitive than others [42].

Figure 2 shows the institutional network diagram of the COVID-19–related information released in February 2020. The connection between nodes represents the simultaneous release of COVID-19–related information by these institutions. The width of the connection line represents the frequency of coreleases, and the wider the line, the more simultaneous the releases. The bold line between G and C indicates that the government and commercial area released the most information simultaneously, followed by the government and educational sector, and then the commercial and educational sector. Within the framework of the quadruple helix model, the government and the educational and commercial institutions were the leading producers of COVID-19–related information, and they played a prominent role.

As seen in Figure 3, April’s COVID-19 messages were primarily from the government and the educational and commercial sectors, and relatively little information was provided by the nonprofit sector, as was the case in February. The number of countries and regions that received information from all four areas simultaneously was the largest. The areas in which COVID-19 information was released in these countries were relatively diverse, including mainland China, the United Kingdom, Brazil, and Japan, with Asian countries accounting for less than half. This is significantly different from the situation in February because, at this stage, COVID-19 became a pandemic and was no longer concentrated in Asia. Information from Romania, Peru, and Chile was primarily from the government and the educational and nonprofit sectors. In Spain, information was mostly from the government and the commercial and nonprofit sectors. Portugal, Italy, India, and Ireland received information primarily from the government and educational sector. Countries where commercial and nonprofit agencies released more information included Israel, the United States, Austria, and Sweden. For South Korea and Poland, government and commercial sectors released more information. In Russia and the Netherlands, most information was shared by government agencies, while in Switzerland and Belgium, educational institutions were the primary sources of COVID-19–related information. In France, the information was primarily shared through the commercial sector. In general, the online information shared about COVID-19 during April and February was quite different in terms of both countries and institutions. Non-Asian countries diversified their fields as COVID-19 became a pandemic.

Figure 4 shows the institutional network diagram of the COVID-19–related information released in April. The government and the educational sector released the most information at this time. The relationship between the government and commercial sector, and that between commercial and nonprofit organizations was also closer. In April, the government and the educational and commercial institutions were still the leading producers of information, playing a prominent role in information dissemination.

The two-mode diagram of the COVID-19 information release in July shows that most countries were delivering information from a diverse range of sectors (Figure 5). The proportion of individual areas and of countries and regions that received information from only two areas was lower than that in the previous phases. The countries and regions that received information from all four sectors the most included mainland China, Russia, Turkey, and the Philippines. In April, the COVID-19 pandemic continued to spread around the world, and the geographic distribution of information was also seen globally, and not just in Asia. Information from Iraq was primarily from the government and commercial and educational organizations. Information in France was from the government and nonprofit sectors. Government agencies in Chile and Italy provided relatively more information. In the United States, information from the government and educational sector decreased, while information from commercial sectors increased. Less information was collected from Bing in the United States in July. Most regions of the world and many industries were affected by the pandemic in July. The structure of the network for information-publishing organizations also developed from the coexistence of double, triple, and quadruple helices to the main structure of quadruple helices.

Figure 6 shows the institutional network diagram representing the COVID-19–related information released in July. The number of concurrent announcements made by the government and commercial sector remained the highest, followed by the government and educational sector, and then the commercial and educational sectors. In the three stages, the government and the educational and commercial institutions were the leading producers of information and played a prominent role.

**Figure 7:**
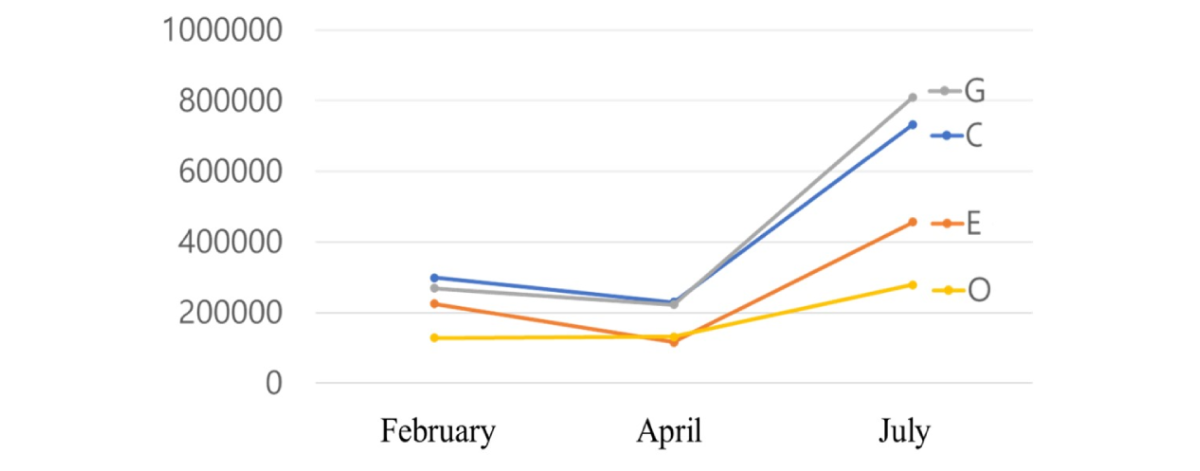
Degrees of the three stages. G: government domains; E: educational institute domains; C: commercial domains; O: nonprofit organization domains.

We collated the degree centralities in four helices and found that the commercial sector in February had the highest degree, followed by the government and educational sector, and finally the nonprofit organizations (Figure 7). In April, the biggest area of degree centrality was again the commercial sector, followed by the government, nonprofit organizations, and finally educational organizations. In July, the government ranked first, commercial organizations ranked second, educational organizations were third, and the nonprofit sector fourth. Thus, the government and commercial organizations played a significant role in the COVID-19 information network, whereas the role of the nonprofit sector was relatively small.

We performed a text analysis and CONCOR analysis for the content of the information shared. For the content analysis, we deleted non-English and scrambled characters during data cleaning. There was a total of 9149 documents in February, and 8889 remained after cleaning. There were originally 14,768 documents in April, 14,766 of which remained after cleaning. The number of documents in July was 14,484 and 13,087 remained after cleaning. Word preprocessing was first performed using Python (the Spacy package) and the results were manually collated. We identified the 50 most frequently found words during each of the three months, which are compiled in Table 4.

Table 4 indicates that in February, people paid the most attention to the affected areas (China), health, departments, international, and news. In April, the content was focused on information regarding deaths, health, the pandemic, and public. In July, the content was focused on information regarding the pandemic, health, and news. These remained the top concerns in July 2020, whereas words such as “online,” “service,” “university,” and “government” were also highly ranked at this time. To summarize, the main content in February was dominated by information and news about the outbreak; in April, information was primarily regarding the public and the pandemic; and in July, various online services were used to address the problems caused by the pandemic.

**Table 4:** The 50 most frequent words of the three stages.

Rank	February			April			July	
	Words	Standardized frequency	Words		Standardized frequency	Words		Standardized frequency
1	coronavirus	100.00	coronavirus		100.00	coronavirus		100.00
2	country	59.30	information		24.96	information		36.79
3	China	30.18	die		21.70	pandemic		24.33
4	novel	28.57	health		12.09	health		22.67
5	health	27.90	virus		9.96	online		18.62
6	Jan	21.14	pandemic		8.22	service		17.20
7	international	19.88	public		7.64	virus		14.21
8	year	19.53	update		6.88	university		12.67
9	department	17.85	country		6.69	government		12.24
10	news	16.35	pour		6.62	news		12.12
11	world	16.00	school		6.60	provide		11.88
12	school	15.91	service		6.58	case		11.28
13	new	15.24	meet		5.91	July		10.49
14	visit	14.22	spread		5.69	new		9.98
15	statement	13.62	April		5.53	development		9.73
16	spread	13.30	government		5.24	education		9.46
17	patient	12.65	March		5.16	ministry		9.46
18	NSW	12.44	SARS		4.92	update		9.46
19	case	10.78	China		4.91	June		9.43
20	epidemic	10.37	situation		4.86	world		9.22
21	response	9.04	help		4.52	country		8.98
22	Japan	8.92	case		4.51	China		8.49
23	student	8.92	work		4.51	support		8.49
24	find	8.81	student		4.48	work		8.31
25	official	8.81	new		4.47	help		8.28
26	category	8.72	continue		4.36	business		8.19
27	university	8.52	provide		4.36	time		7.83
28	staff	8.40	novel		4.13	public		7.59
29	information	7.98	outbreak		4.13	spread		7.35
30	symptom	7.87	community		4.01	March		7.29
31	child	7.43	care		3.91	student		7.29
32	like	6.96	support		3.91	include		7.26
33	continue	6.82	online		3.88	Pakistan		7.10
34	city	6.71	people		3.75	SARS		7.04
35	public	6.67	website		3.75	India		6.86
36	Feb	6.59	maatregelen		3.67	Indonesia		6.77
37	ministry	6.12	include		3.64	community		6.71
38	disease	5.98	medidas		3.53	website		6.56
39	big	5.97	time		3.52	year		6.50
40	monitor	5.95	business		3.38	measure		6.47
41	pneumonia	5.83	impact		3.23	continue		6.44
42	update	5.75	man		3.11	disease		6.41
43	government	5.36	find		3.08	national		6.41
44	service	5.33	university		3.07	people		6.35
45	unfold	5.07	page		3.06	find		6.32
46	view	4.81	contact		3.02	medidas		6.32
47	Singapore	4.63	disease		2.98	home		6.26
48	cause	4.60	staff		2.94	social		6.14
49	infect	4.58	late		2.93	South		6.14
50	Chinese	4.29	measure		2.92	read		6.08

To further assimilate valuable information, we constructed the semantic network of high-frequency words and used CONCOR analysis for clustering. Furthermore, we obtained a visualization diagram of the clustering network in the three stages.

**Figure 8:**
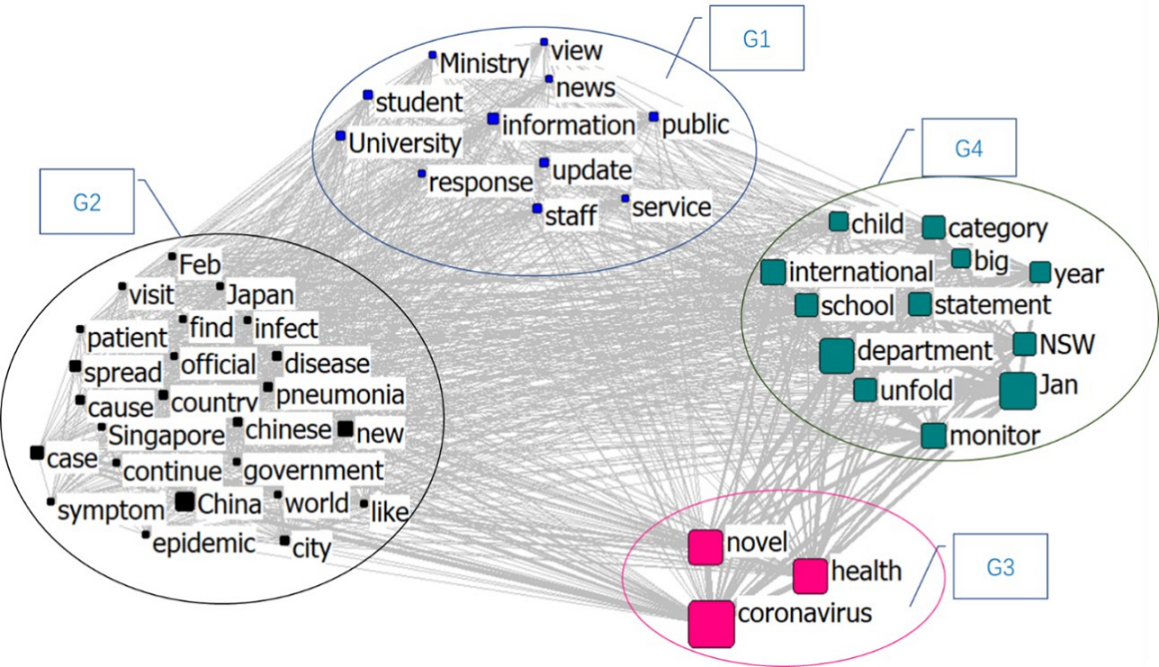
Semantic network in February 2020.

Group 1 in Figure 8 is named “Ministry and University,” which contains information on services provided by the government departments, public authorities, and schools. Group 2 is named “Virus Spreading,” which primarily includes confirmed cities, patient symptoms, and the spread of the infection. Group 3 is “Coronavirus and Health” and Group 4 is “Child and Education.” Most of the information in Group 4 is related to “NSW (New South Wales, Australia) education” and “child,” and it showed the highest frequency in February.

**Figure 9:**
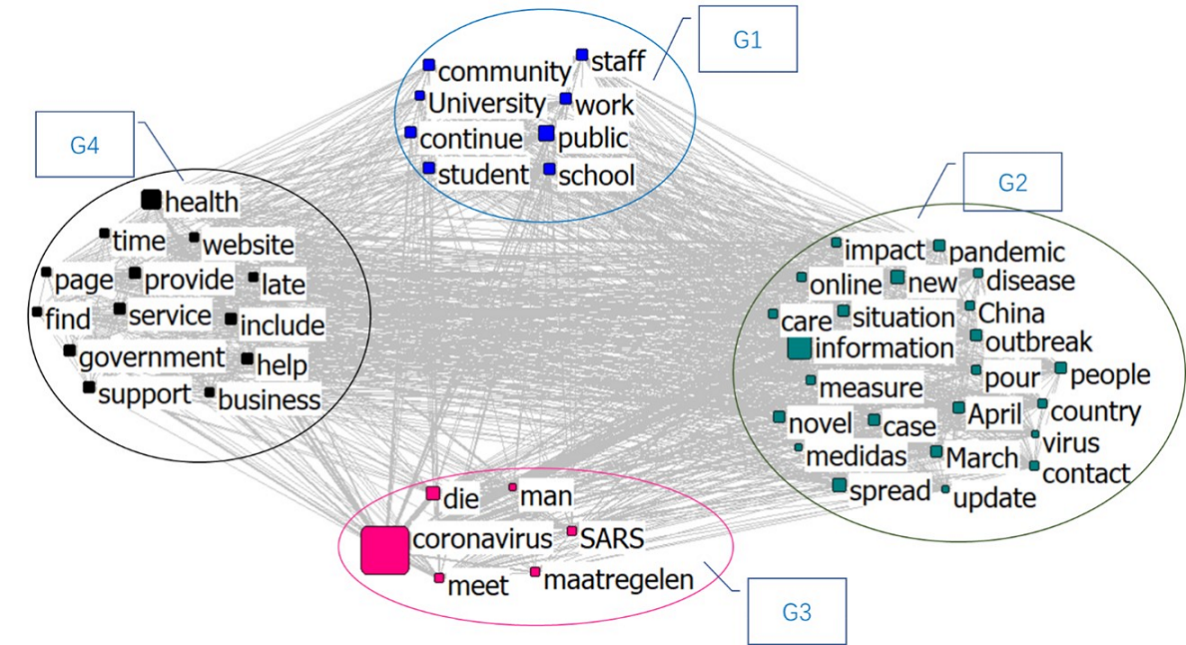
Semantic network in April 2020.

Figure 9 shows the semantic network for April. Group 1 is a school and student-related group named “Education Issue.” Group 2 is “Virus Spreading,” including information about the outbreak and the spread of the pandemic. Group 3 is “Virus Description,” which contains information related to the characteristics of the virus. Group 4 is “Commercial Issue,” which includes words such as “business,” “government,” “service,” and “help,” and is related to the social change brought about by the pandemic.

**Figure 10:**
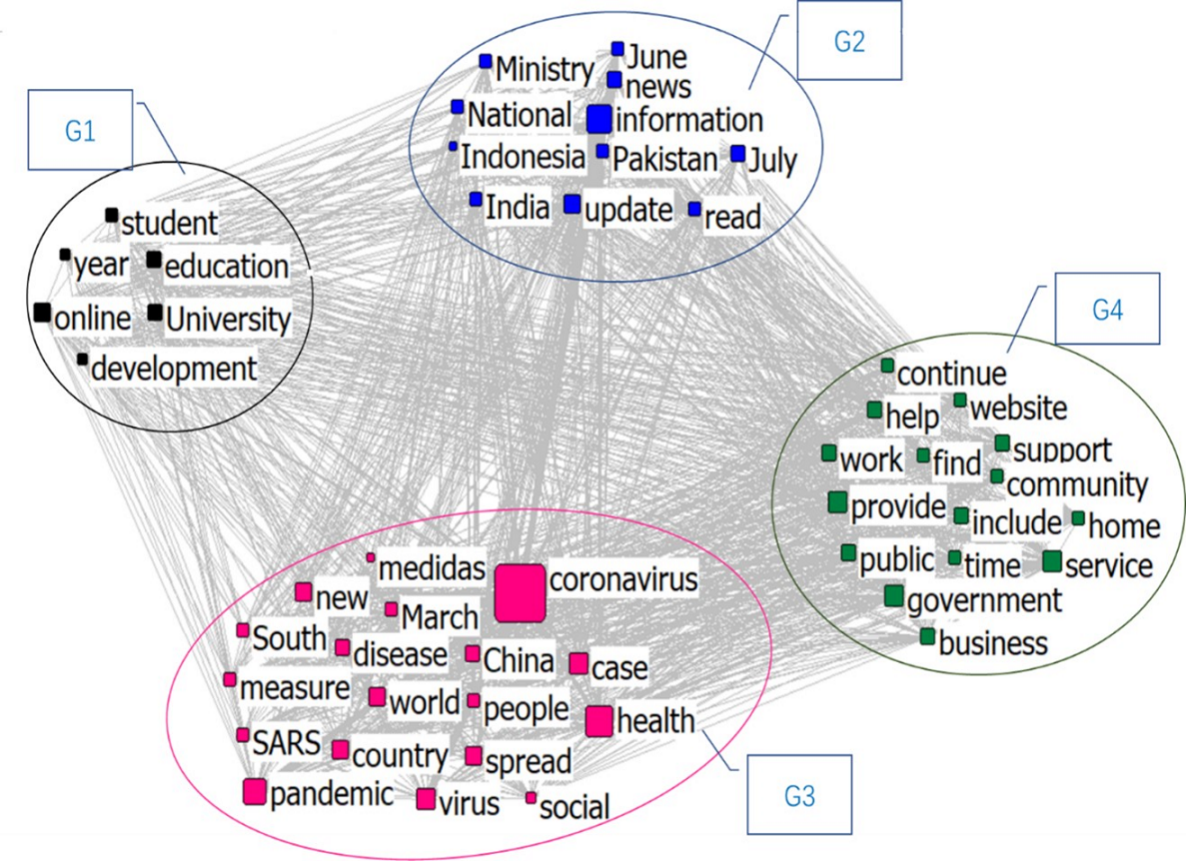
Semantic network in July 2020.

In July, the semantic network was also divided into four groups, as shown in Figure 10. Group 1 is “Distance Education,” which contains information about online education. Group 2 is “City News,” which contains information about the cities affected by the pandemic. Group 3 contains information about the measures taken, and is therefore named “Measures.” The last group is “Commercial Issues,” which includes information regarding “business,” “services,” “government,” “provide,” “community,” and similar.

**Figure 11:**
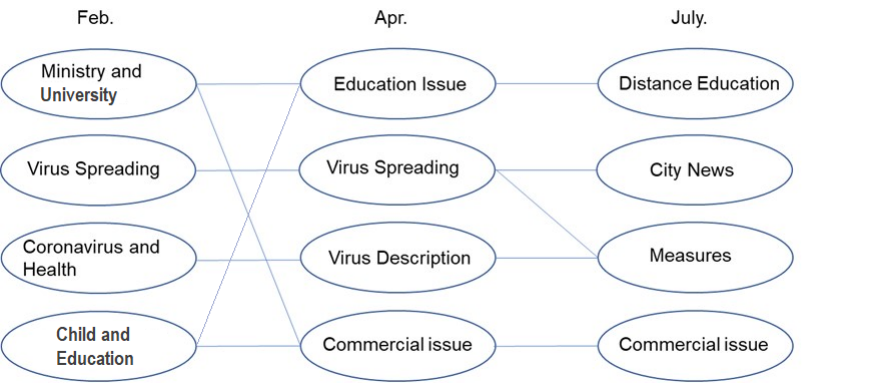
Evolution of topic content.

The clustering topics of the three stages are sorted in Figure 11. The results show that the information in February was mainly regarding the response of the government and educational institutes, such as the impact on schools after the virus began spreading, and the spread of the virus and health issues became prominent topics. In addition, since the spread of COVID-19 was at an early stage in February, information reports in some places were also relatively prominent. By April, the disease had become a pandemic. At this stage, besides information, the spread of the virus, the description of the related characteristics of the virus, and the changes of the commercial environment became prominent topics. By July, the focus in education shifted to distance education. As the pandemic could not be fully controlled within a short period of time, most educational institutions began to prepare for or implement online education. By this time, the public had a basic understanding of the virus and how it spreads, and the focus shifted to measures such as how to deal with this spread. The impact of the pandemic on business and society was still an important topic. Information about cities related to the outbreak also continued to appear in the news.

In general, education became a prominent topic of discussion in all three stages. With time, the basic information regarding the virus and its transmission became popularized, and people began to pay more attention to information about measures to prevent its spread. Since the beginning of the pandemic, the situation has changed in terms of business, government policy, and other public issues. Society has also changed. We compared the results of content analysis with the results of the quadruple helix structure and found that the content analysis also confirmed the form of the quadruple helix structure. In the content analysis, the information groups about business issues and government emerged as relatively large, with a smaller contribution of information about education, although this topic also forms a certain scale of the groups.

## Discussion

### Principal Findings

In this study, we analyzed COVID-19 web information sources from a quadruple helix perspective, and found changes in structure and content at each stage during the first 6 months of online information regarding COVID-19. We also found problems in the structure of information sources in the transmission of relevant information. We here provide detailed suggestions, which can contribute to the internet communication of future public health events.

Based on the quadruple helix model, this study collated and analyzed the structure and content of the network information sources about COVID-19 considering time and space. By sorting out the second TLD, we divided the structure of network information sources into four categories: the government, education, companies, and nonprofit organizations. An information source network composed of four levels was obtained. The results of the two-mode quadruple helix analysis of the three stages showed that the major confirmed cases in February (first stage) occurred in Asia, and the online information sources in Asia were more diversified than those in other regions. As the pandemic spread in April (second stage), non-Asian sources of information began to diversify, and in July (third stage), the sources of web information became globalized. Thus, the impact of the first stage of the pandemic was more sensitive in Asia, and the information from various industries was related to responding to this need. However, only some industries in non-Asian regions paid attention in the first stage, and the information source helix did not form, which also led to the slow response to COVID-19 in some regions and the delay in response measures [43]. Since April, the spiral has intensified in non-Asian regions due to the spread of the pandemic to many areas outside Asia, which has raised concern of various industries.

In general, from the results of the two-mode analysis, the structure of the three stages of web information publishing organization has gradually developed from the coexisting structure of a double, triple, and quadruple helix to the diversified structure centered on the triple and quadruple helix. From this phenomenon, we can find that in the face of major public health emergencies, most of the local information release sources are not comprehensive. This phenomenon has also led to the failure of many industries to anticipate and respond to the pandemic in a timely fashion [44,45]. Our results suggest that the health care sector can call on the local information sources of various industries to release appropriate and reasonable information about the health and public events in the future to ensure the timely deployment of all sectors of society and avoid more losses. We used a modular quadruple helix structure to analyze the forces of these four levels at various periods in detail. We found that in February, the information shared was the most coincident and closely linked between government and commercial organizations, followed by educational and government organizations. Next, there was a closeness between the commercial and educational sectors. In April, the government and the educational sector simultaneously released the most information about COVID-19. The relationship between the government and commercial sectors, and the relationship between commercial and nonprofit sectors were also closer. In July, the number of concurrent announcements about COVID-19 by the government and commercial sector remained the highest, followed by the government and educational sector, and then the commercial and educational sectors. We collated the centrality of the three stages and four areas, indicating that the commercial area scored the highest in February, followed by the government and educational sectors, and finally the nonprofit organizations. In April, the biggest area of degree centrality was also the commercial sector, followed by the government, nonprofit sector, and finally educational enterprises. In July, during the third stage, the government played a central role in the COVID-19 information network. In all three stages, as a whole, the government and commercial sector played a significant role in COVID-19 network information, and the connection of the nonprofit sector was relatively low. In fact, in the event of major infectious diseases, school is an important aspect that cannot be ignored, and schools often gather dense populations [46]. The communication role of the education sector as an information source is not stronger than that of business and government sectors. However, as educational institutions learn more than any other institutions about the actual school and education situation, they should take on more of a role than the government and businesses to ensure the spread of information. In future infectious disease health events, education and industry organizations, along with others, need to release information more quickly and accurately.

This study included an analysis of the quadruple helix structure and the content of the three stages using dynamic progressive detailed analysis. We carried out content analysis on 36,742 pieces of information in the three stages. The results of frequency analysis showed that the most prominent information in February was news about the pandemic. April was dominated by information about the public and the pandemic. The focus in July was the use of various online services to solve problems caused by the pandemic. We then used CONCOR cluster analysis to classify the topics in the three stages. The changes in trends in the three stages were also sorted. The results indicated that in the early stages, there were more reports about the affected areas, and the response of authorities such as governments and schools to the virus, and the spread of the virus and health issues were the main points of focus of discussion. The second phase focused on the spread of the virus across the world, which created a global pandemic. At this stage, information about educational hotspots, descriptions of virus-related features, and information about commercial environment changes caused by the pandemic also received attention. In the third stage, the educational hotspots differentiated into the characteristics of distance education. The pandemic made physical face-to-face education difficult. Many educational institutions began to prepare for or implement online education. Public attention at this stage shifted from what the virus was to measures of controlling its spread. In general, education was a prominent topic at all levels. With the change of stage, the information content also changed. In the early stage, the basic situation of the virus and its impact on health attracted most of the attention. Later, the focus was on pandemic prevention measures. The business environment and policy environment have changed from the beginning of the pandemic, and the social changes caused by the pandemic have also become an important discussion topic.

### Limitations

Owing to the large amount of data from all countries worldwide, this study has only used the web information for countries with a significant number of diagnosed cases at each stage as the research object. In addition, we only used data from Bing. Although Bing is more widely used than any other search engine in the webometric field, it does not have a strong market share in some parts of the world that rely more on other search engines. For example, Google has the largest market share in the United States, Baidu has the largest share in China, and Naver has the largest share in South Korea. Therefore, the results of different search engines in individual regions may somewhat vary from those of Bing. In addition, there is no ideal description of the web network structure [47]. Search engine properties are considered as more engineering products than mathematical tools [48]. Different search engines often have divergent algorithms and search results, which inevitably produce repeated and mixed results. Since search engines usually consider both quality and efficiency, this could also lead to problems related to Type I and Type II errors, which objectively lead to insufficient coverage [48,49]. These can be considered as limitations of the study.

### Conclusions

This study focused on the structure of information sources at each stage of the first 6 months of the COVID-19 pandemic and the development of the network structure through the quadruple helix framework. We found that for public health emergencies, some online and offline information sources were not sufficient. Diversified institutions need to pay attention to public health emergencies, and actively respond to multihelical information sources, which is conducive to implementing a timely and more comprehensive response to public health emergencies. In terms of published messages, the educational sector plays an important role in public health events. However, educational institutions release less information than governments and businesses. In addition, we summarized the trend of COVID-19 online information dissemination. It is important to understand the communicational structure of pandemic information sources worldwide. Currently, the quadruple helix model is primarily used in the field of scientific cooperation in terms of coauthorship analysis, and research in other fields is insufficient. This study highlights that the quadruple helix not only has theoretical significance in the scientific innovation field but can be also used to conduct effective research regarding web information. This is significant for further development of the quadruple helix model with respect to the COVID-19 pandemic.

## Abbreviations

CONCOR: convergence of iterated correlations
TLD: top-level domain
